# Monthly Summary

**Published:** 1875-09

**Authors:** 


### MONTHLY SUMMARY.

*Death from Ether Inhalation.*—Unfortunately, even ether, it seems, is not to be inhaled always with impunity. A boy, aged 16, was operated on by Dr. Hardie, of Manchester, at the Workhouse Infirmary, on the 3rd of April, 1875. Four drachms of ether were poured on a piece of lint and placed in a folded towel, which was held pretty close to his face. There was no coughing, and but little struggling. He was ready for operation in about four minutes without any more ether. The respiration suddenly ceased, and the pulse at the wrist became imperceptible. The tongue was pulled forward and the poles of a battery applied to the phrenic nerves. Cold water affusion, artificial respiration and suspension by the feet were tried, without avail. The organs were found healthy. The cavities of the heart were empty. Probably death arose from syncope. Robbin's ether was used.

This is a most untoward event, because it may prevent the growing tendency to use ether instead of chloroform from spreading as it ought. Ether is certainly far safer and ought therefore always to be used instead of chloroform.—*The Doctor*.

---

*Poisoning by Aconite.*—Dr. J. E. Blake, in an interesting article in the *New York Medical Journal*, relates a very important case of poisoning by aconite and chloroform liniment. An hour after the poison was taken, "*no pulse could be detected even in the axilla, and she remained without any trace of pulse for a period of over three hours.*" Nevertheless, Dr. Blake persevered all day and all night with galvanism and artificial respiration, and was rewarded by complete success. He had previously, within half an hour of the poison being swallowed, used the stomach-pump until fluid returned unimpregnated with it; but enough had been absorbed to do deadly work. This patient became early *unconscious*, and remained so for many hours. Yet almost all writers assert that in aconite poisoning *consciousness* continues to the last. Could the chloroform account for the unusual fact? The lady had taken "a little more than a drachm" of the liniment, which consisted of chloroform and tincture of aconite root in equal quantities.—*The Doctor*.

*Carbolic Acid as an Antiphlogistic.*—Hagen (*Aerztl Intellig. Bl.*) reports several cases of phlegmonoust inflammation of the hand, croupous laryngitis, and croupous pneumonia, which he has cured by injections of a solution of carbolic acid in the proportion of 2,100 (one to two syringeful daily in adults, and half a syringeful in children.) Even after the first injection the fever, pain and swelling, were greatly modified. The injections were always made in the vicinity of the affected part. Kunge likewise reports happy results from this treatment in acute articular rheumatism and pneumonia.—*Med. Chir. Centralblatt.*—*N. Y. Med. Journal*

---

*Physiological Experiment Upon the Human Corpse.*—The experiments made by Drs. Keen and Seiler upon the body of Heidenblut, immediately after its removal from the gallows, showed that the internal intercostals are muscles of inspiration, and the external intercostals muscles of expiration; the former lifting the ribs, the latter depressing them. In testing the facial muscles, it was also shown that the pyramidalii nasi is a direct antagonist to the occipito-frontalis.—*Nashville Medical and Surgical Journal.*

---

*Toothache.*—Dr. Q C. Smith praises the following most highly: Take of carbolic acid saturated solution, chloral hydrate, saturated solution, paregoric, fluid extract of aconite, of each an ounce; of oil of peppermint half an ounce; saturate the pledget of cotton or a piece of sponge, and tightly pack in the cavity. *Charleston Med. Jour. and Review*, April, 1875.

---

*Location of the Sense of Taste.*—According to Dr. Davidson, of Liverpool, the results of clinical observation, and of physiological experiment appear to show conclusively that the sense of taste in the anterior area is dependent on the integrity of the chords tympani, since injury to the fifth nerve, above the junction of the latter with it, although accompanied by complete anæsthesia of the side of the face and tongue, does not necessarily affect the sense of taste; whilst, on the other hand, when the chorda tympani is injured, or cut, taste is lost without loss of sensation. According to some, who regard the chorda tympani as a purely motor nerve, this is due to the affection of the functions of the submaxillary gland, or to the loss of power of erection of the taste papillæ. Neither of these explanations, however, appear to be satisfactory, and the weight of evidence would seem to be in favor of the view that some of the fibres of the nerve are afferent.—*Med. & Surg. Reporter.*

*Efficiency of Mineral Water in Reducing and Adding Fat.*—Perlet, the well known actor, whose leanness is described as "something phenomenal," naturally desiring to get "some flesh on his bones," a well-known physician of Paris advised him to go to one of the bathing places in the Pyrenees. Perlet accordingly went off to the prescribed locality, where he drank and bathed with the utmost zeal and perseverance. But neither drinking nor bathing seemed to have any effect upon him, and he remained just as much a skeleton as before. "Patience!" urged the local doctor, in reply to his expressions of disappointment; "there is nothing like the water of our springs for making people fat!" Soon after Perlet was patiently soaking himself in a bath, and heard a colloquy in the bathing cabinet next his own between the local Æsculapius and a lady of enormous obesity. "Doctor," remarked the lady, "I am really losing heart and patience." "Why so?" inquired the doctor. "Because, though I have been taking these waters regularly for two months, I am not an ounce lighter?" "Patience, madam!" said the doctor, in his most persuasive tones, "*there is nothing like the water of our springs for making people thin!*" —*The Doctor.—The Clinic.*

---

*A New Cause for Blue (Lead) Line on Gums*—A writer in the London *Lancet* asserts that the constant use of powdered charcoal as a dentrifice will produce a blue line on the gums closely simulating that of lead-poisoning.

---

*Wine Drinking and Drunkenness.*—Again we have it stated, this time by Dr. Lunier, in a paper at the Paris Academy of Sciences, that drunkenness becomes more general in proportion as the alcohol of commerce is introduced into common use. Thus, in wine-producing countries, where wine is the ordinary drink, drunkenness is rare; whilst, on the contrary, it is general in countries which do not produce wine. His remedy for this state of things is to give every possible facility for the consumption of wine in those countries where it is an imported luxury. —*Med and Surg. Reporter.*

---

*A New Mode of Burial* is recommended by a Vienna chemist. It consists in placing the corpse in a coffin of cement and then filling in around it with the same material made plastic by the addition of water. This is then covered closely by a cemented cover, thus preventing all putrefaction, and the absorption of the fluids of the body by the cement would soon mummify it. A coffin of this kind would cost but little more than an ordinary wooden one, and as the filling soon hardens, these coffins can, if required, be piled in tiers one upon the other.—*Clinic.*

*Over-work or Over-play.*—The question as to what amount of work can be performed by each patient must come daily before the medical man, and he is forced to answer it. The question is generally put by those who have already made the assumption that they are overworked, and therefore are willing to receive but one answer. Apart, however, from prescribing in accordance with the wishes of our patients, the subject is important, and one fruitful of discussion. The amount of work which each man or woman is capable of performing is dependent on their temperament and especial powers, and can only be gauged by their individual capacities. There are some young men whose brains are so weak that they cannot bear the pressure which is put upon them by a little sustained thought. Every year I see one or two medical students obliged to leeve their chosen profession and devote themselves to an out-of-door active life. They are quite unable to apply themselves to studies which required any concentration of mind, and at last they throw them up. I have seen the same in those who have intended to follow the law, and more than one banker's clerk who could not bear the drudgery of the ledger. Whilst writing these lines I have heard a young man, a Government clerk, speak of over-work, although his office hours are only from nine o'clock until three. To discover the amount of brain-work proportionate to each individual lad is a difficult task ; but the cases I have mentioned show that there are persons who cannot sustain any mental tension. At the same time there are those in business, and the more the pity, who would have adorned any of the professions. I have known more than one mind made in a rare scientific mould entirely wasted. Apart, however, from these individual cases, which must be judged on their own merits, I am anxious to know whether medical men generally accept what seems to be a very popular opinion of the day, and which has been thought to be expressed in Mr. Greg's lecture, that society at large is really suffering from an amount of work, physical and mental, which is injurious to the individual, and therefore to the human race. That an opinion of this kind largely prevails I gather from conversation, and from the public prints; but lately I saw quotations from the sermon of a well known preacher, in which he declared that our asylums were filled with mad people, owing to the overwrought state of the nervous system in the present hard-working age. Now, if the question be put thus broadly—Are people suffering from overwork? I, for one, should have no hesitation in saying, No; but. on the contrary, if both sexes be taken, I should say the opposite is nearer the truth, and that more persons are suffering from idleness than from excessive work. Medically speaking, I see half a dozen persons suffering from want of occupation to one who is crippled by his labors.

I have therefore, verv little sympathy with the prevalent notion that nervous and other diseases are due to overwork; and, as regards the statement of the preacher above mentioned, I apprehend he never thought of testing the truth of it by a visit to the public asylums, for he might then have been impressed with the sight of the numbers of the lower classes and the altogether unlettered persons confined therein.—*Dr. Samuel Wilkes, in London Lancet.*

---

*A Calculus of Steno's Duct, having for its Origin a Grain of Wheat.*—At the same meeting, M. Paquet reported the case of a man, fifty-two years of age, who had repeated attacks of inflammatory swelling of the cheek and molar region. The abscess had already opened several times into the mouth, before the patient came for treatment. The surgeon found a swelling of the duct of Steno, which was ulcerated at its edges, and he was able, by means of a curette, to extract from its orifice an ovoidal flattened calculus, weighing one gramme, which was followed by the escape of a large quantity of pus. Injections were made into the seat of trouble, and a rapid cure was completed. The examination of the calculus presented an interesting point in regard to etiology, for in its centre was found a grain of wheat.—*Med. and Surg. Reporter.*

---

*Dangerous Compound.*—*The Pharmaceutical Journal* of Vienna states that the following prescription was sent to a pharmacist: Chronic acid, eight grains; glycerine, one drachm. For external use. The dispenser dissolved the acid with a little water in a phial by a little shaking; the glycerine was then poured in, and the phial again shaken. Thereupon the compound exploded with a very loud report, and was projected with force to the ceiling of the shop. The phial which did not break, became coated with a black pigment, and remained in the hand of the frightened dispenser. The case deserves attention from the fact that the ingredients were in such small quantities and the explosion so powerful.—*Med. Record.*

---

*A British Dental Association.*—The last number of the *Monthly Review of Dental Med. and Surg*, contains a proposition for the formetion of a British Dental Association. It is proposed to assimilate its character and constitution to those of the British Medical Association, and to hold the meetingc at the same time and place as those of the latter.

*An Advanr in Dental Surgery.*—Before "The American Academy of Dental Surgery," held on Thursday evening, June 10, 1875, George H. Perine, D. D. S., of New York, made some interesting remarks on the use of the galvano-cautery for oral surgery, which were followed by illustration.

Dr. Perine claims to be the first person who has applied that particular cautery in dental surgery. He has found no agent so available and so efficacious in certain dental operations as the galvano-cautery, and strongly recommends its use to the profession at large. The battery employed by Dr. Perine is one of great power, and is regulated to suit the operation. The advantages claimed are that it does not affect the body in any degree unpleasantly, because the electric action is absolutely confined to the parts in contact with the blade or broach, the application of which is painless, and so instantaneous that no other known agent can compare with it; the application of it is easy and simple, and free from shock and hemorrhage (a desideratum of great moment,) and finally, the separation process is rapid. Dr. Perine not only reasons warmly on this subject, so important, but he believes that the use of this particular cautery in dental surgery will become universal, for the extension of which he cheerfully imparts to his professional brethren his knowledge and experience.—*Med. Kecoad.*

---

*A Large Mouth.*—Dr. Pixton, dentist, of Lancaster, has the impression of the mouth of a colored woman, taken by Dr. Robinson, of Rome, New York. The jaw is three inches from front to back, and three inches across, constituting the largest human mouth known to the dental profession in this country. The ordinary mouth is only 1½ by 2 inches.—*Med. and Surg. Journal.*

---

*A Cure for Drunkenness.*—The American *Journal of Pharmacy* says that in Russia, for some time past, Herba serpylli [wild thyme] has been used with great success to effect a permanent cure of drunkenness; in case of a relapse [only after years,] a short treatment will effect a cure again. The treatment consists in making an infusion of wild thyme [1½ oz to 1½ pints,] and giving the patient a teacupful every half hour. The next day it is given every two hours, and then four to six times a day until the cure is complete, which generally takes from 2 to 3 weeks. The effects are in the following order: vomiting, diarrhœa, increased urine, strong transpiration; then, generally, increased appetite and craving for acidulous beverages. The diet easily digested food, and lemonade or other acidulous liquids.—*Med. and Surg. Reporter.*

